# Optometrist-delivered selective laser trabeculoplasty in the HES – a training protocol and early service evaluation

**DOI:** 10.1038/s41433-024-03086-1

**Published:** 2024-05-03

**Authors:** Evgenia Konstantakopoulou, Jay Varia, Janisha Parmar, Neil Nathwani, Scott Hau, Wing Shing Low, Laura A. Edwards, Emma Laber, Minak Bhalla, Gus Gazzard, Hari Jayaram

**Affiliations:** 1 NIHR Biomedical Research Centre at Moorfields Eye Hospital NHS Foundation Trust and the UCL Institute of Ophthalmology, London, UK; 2 Glaucoma Service, Moorfields Eye Hospital NHS Foundation Trust, London, UK; 3 Optometry Education, Moorfields Eye Hospital NHS Foundation Trust, London, UK; 4 Division of Optics and Optometry, University of West Attica, Athens, Greece

**Keywords:** Health occupations, Therapeutics

## Abstract

Over the last decade, the delivery of glaucoma care in the UK has changed dramatically, with more non-medical ophthalmic practitioners involved in the care of glaucoma patients. Optometrists and other non-medical professionals are now involved in the delivery of laser treatments in the Hospital Eye Service (HES), but there is currently no standardised national training framework for non-medical clinicians. Moorfields Eye Hospital and UCL’s Institute of Ophthalmology have developed and delivered an education and training programme for the delivery of lasers, including Selective Laser Trabeculoplasty (SLT) by non-medical ophthalmic practitioners. The training programme is based on medical education principles, is informed by previous qualitative research into the role of ophthalmic practitioners in the delivery of laser treatments and is expected to have multidisciplinary benefits for ophthalmic healthcare. Clinical audit data indicate that optometrists can deliver safe SLT treatments, adhering to local protocols.

## Introduction

Glaucoma is the leading cause of preventable visual impairment (VI) in the UK [1]. It is a progressive optic neuropathy usually associated with elevated intraocular pressure that requires life-long treatment and monitoring by eye-care professionals and if left untreated can lead to blindness [2].

Current treatments for glaucoma are based on lowering the intraocular pressure (IOP). IOP can be reduced using medications, laser or surgery. Until recently eyedrops were the recommended 1st line treatment but are associated with local and systemic side effects, adherence issues and healthcare costs [3–5]. In 2019 the Laser in Glaucoma and Ocular Hypertension (LiGHT) [5] randomised controlled trial provided evidence to support Selective Laser Trabeculoplasty (SLT) as a primary treatment for primary open-angle glaucoma (OAG) and ocular hypertension (OHT) and has subsequently reformed treatment protocols across the world.

SLT is a quick outpatient procedure for lowering IOP. The precise mechanism of action is not fully understood but it is thought to involve remodelling of the trabecular meshwork, leading to increased aqueous outflow [6, 7]. SLT is as effective as eyedrops in terms of IOP control in eyes with newly diagnosed, previously untreated OAG or OHT, with almost 70% of treated patients remaining drop-free for at least 6 years and has been shown to delay the need for surgery to lower IOP [5, 8]. SLT is also cost-effective for the NHS and possibly for similarly structured fixed-budget healthcare systems internationally [5].

**Fig. 1:**
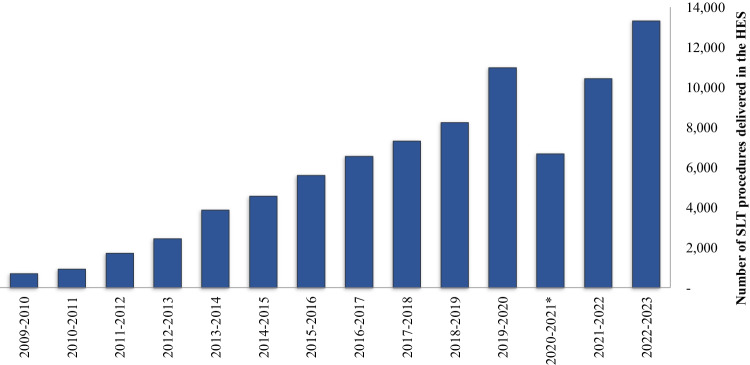
Number of SLT procedures delivered in the NHS HES per year. *2020-2021 data reflect the pandemic restrictions applied to HES visits. SLT selective laser trabeculoplasty; NHS National Health Service; HES Hospital Eye Service.

Following the publication of the LiGHT trial results, the United Kingdom National Institute for Health and Care Excellence (NICE) [9] guidelines were updated, recommending that all new patients diagnosed with OAG and/or OHT should be offered SLT as a primary treatment. This update has also been incorporated within guidance from the European Glaucoma Society [10] and preferred practice patterns from the American Academy of Ophthalmology [11]. Over the last decade, and since the update of the NICE guidelines the number of SLT treatments delivered in the NHS has steadily increased (Fig. 1) [12]. In 2022-2023 a total of 13,323 SLT treatments took place in the HES, indicating an urgent need for appropriately trained healthcare professionals to deliver these treatments, without impacting on the delivery of routine glaucoma monitoring.

The NHS glaucoma services within the UK have been under significant scrutiny in recent years, following cases of severe vision loss attributed to the lack of capacity for timely monitoring of patients [13, 14] with further delays attributable to delays from cancellations due to the COVID-19 pandemic [15]. In response to these incidents, a search for new, efficient and safe models of care has commenced, including the management of glaucoma by appropriately trained non-medical professionals [16–19].

Hospital optometrists are involved in nearly 90% of glaucoma clinics nationwide [20]. They assess and manage patients with glaucoma in the HES according to current NICE guidelines (NG81) [9], as well as in community-enhanced care schemes, and their training is underpinned by the College of Optometrists professional higher qualifications (Professional Certificate in Glaucoma, Higher Certificate in Glaucoma, Diploma in Glaucoma) or the Royal College of Ophthalmologists (RCOphth) Ophthalmic Practitioner Framework (OPT) [21, 22]. Two workforce surveys by Harper et al. [23] and Gunn et al. [20] indicate that glaucoma care is the most common extended role provided by hospital optometrists with 84% of respondents practising predominantly independently [24]. The latter survey indicated that up to 2020 optometrists regularly delivered SLT in 14 glaucoma units in the UK compared to 1 unit in 2016 [20]. Despite this significant increase in non-medical delivery of SLT, there is yet no formal laser training for the delivery of laser procedures by ophthalmic practitioners, with apprenticeship style training varying between units in the country; similar training standardisation issues are also present in the US [25].

The lack of standardised quality-assured laser training in the UK also extends to ophthalmology in vivo training. The RCOphth encourages ophthalmology trainees to practice on model eyes before performing laser procedures [26], but trainees must work under clinical supervision to progress to independently providing laser treatments such as SLT. It has been previously suggested that a structured laser-training programme for optometrists could provide a common basis for standardised training for trainee ophthalmologists [27].

Building from previous work on the global landscape of non-medical delivery of SLT [25] and the acceptance of optometrists in such roles [27], here we report the principles and delivery of a laser course for non-medical ophthalmic clinicians delivered by Moorfields Eye Hospital NHS Foundation Trust and the Institute of Ophthalmology at University College London (UCL). The training course is designed for optometrists following established glaucoma training, underpinned by the College of Optometrists Higher Qualifications curricula [22] and the RCOphth OPT curriculum [28]. We report initial data on the performance and safety of optometrist-delivered SLT following the training and the impact on services. Detailed data on the clinical -efficacy of optometrist-delivered SLT will be published in the future.

## The training programme

The programme was designed by Moorfields Optometry Education to comprehensively train ophthalmic healthcare professionals to competently deliver SLT including pre- and post-treatment care, underpinned by sound educational principles. The course provided structure, adding elements to the existing in-house laser training at Moorfields, and was open to all external ophthalmic clinicians. The course aimed to allow clinicians to understand the basic principles and evidence-based indications of ophthalmic therapeutic laser applications, enabling them to perform the procedure with effective guidance on appraisal and safe application to clinical practice, whilst incorporating basic principles of consent and post-laser patient management, including management of complications. The programme was funded by Moorfields Optometry Education.

### Entry requirements

Entry requirements for the course were a minimum of a BSc in Optometry, Orthoptics or Nursing with ophthalmic speciality training equivalent to OPT level 3, the College of Optometrists Glaucoma Higher Certificate or an equivalent qualification and experience, a certified gonioscopy competency and independent prescribing qualifications (or evidence they were supplementary prescribers or could prescribe under a Patient Group Direction). These entry-level criteria were based on previous qualitative work gathering the views of consultant ophthalmologists and optometrists on the development of a laser-training course for non-medical professionals [27]. The criteria were also supported by evidence that the clinical decision-making of optometrists with higher qualifications or experience in specialist HES clinics is comparable to consultant ophthalmologists [29–31] and better than peers without such qualifications [32].

### Stage 1: Knowledge acquisition and demonstration of procedure

#### Online asynchronous lectures

Online lectures covered the theoretical component of the course and were developed by specialist glaucoma ophthalmologists and optometrists who regularly perform laser procedures at Moorfields Eye Hospital. This covered basic laser principles, laser safety and risks, contra-indications, complications, evidence base, medico-legal aspects and consenting for laser. Learners had opportunities for peer discussion and assessments, testing recall. A summative pass/fail assessment concluded this section; a pass was required for progressing to the next stage.

#### Practical training day

Hands-on clinical training was delivered using specialist model eyes designed for SLT. Each delegate had the opportunity to observe the delivery of SLT in a model eye, followed by hands-on experience, delivering SLT to 360^o^ of this eye. Each delegate delivered SLT to 360^o^ of a model eye, with one-to-one supervision by an expert clinician. Following the clinical training session, delegates had a short period of reflection. Formative assessments in the delivery of SLT using model eyes followed using a Direct Observation of Procedures (DOPs) form, where learners were given individual feedback on the preparation of the instruments and the patient, safe and correct delivery of the laser, understanding of the risks and potential complications and their management. Case studies were also presented and discussed. Learners were given a course education pack with guidance and forms enabling them to proceed to the next two stages of training in their workplace.

### Stage 2 – work-based learning and assessment

This stage was carried out exclusively in the clinicians’ workplace and involved work-based assessment competency sign-offs, carrying out supervised laser procedures ‘in vivo’. Competency sign-off was done using adapted DOPs forms, where learners received detailed feedback from their assessors who were experts in the delivery of SLT. Learners were also expected to discuss cases with their supervisors and were formatively assessed using a standard Case-based Discussion (CbD) form. Optometrists were recommended to observe ten [10] procedures and to deliver another ten procedures [10] under supervision, before completing a minimum of another fifty (50) eyes independently; this was a tentative guide given the lack of an evidence-based prescribed number of procedures needing to be completed [27, 33]. It was recognised, however, that the final sign-off would be given by a clinical supervisor when satisfied that the learner was safe and although the minimum was fifty eyes, this number could be exceeded.

### Stage 3 – reflective practice

#### The self- audit

As part of their training, optometrists were provided with training on audit principles and were advised to perform a self-audit after the first 50 SLT treatments delivered, against set criteria such as prescribing, complications and success rates; these criteria were provided in the SLT training pack. Criteria were not limited to this, and the clinicians’ employment organisations could add to this.

#### The reflective statement

**Fig. 2:**
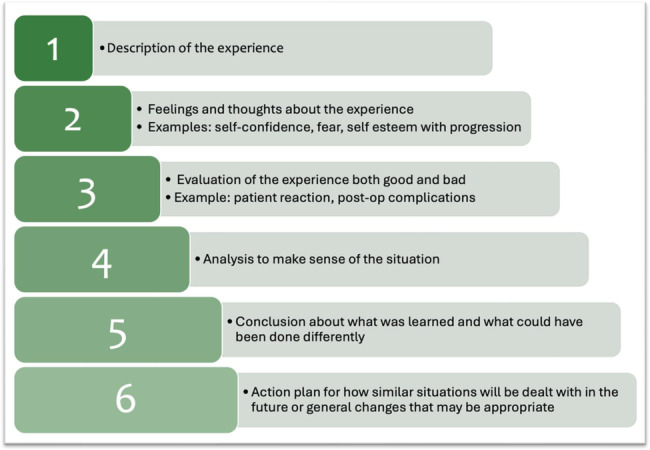
Guidance for learner’s reflective statement based on underlying principles of Gibb’s reflective cycle, adapted from underlying principles of Gibb’s reflective cycle. These enabled learners to structure their narrative, leading them through six stages when exploring their experience: describing their expertise and building critically on this to articulate feelings, evaluate and analyse their experience, culminating in conclusions and action plans.

Clinicians were asked to write a 1000-word reflective statement at the end of their training, discuss the issues that they felt needed to be addressed or discussed with their workplace supervisor and potentially disseminate or share findings with others. Guidance for the reflective statement was included in the packs and was based on principles underpinned by Gibb’s reflective cycle [34] (Fig. 2). The aim of this stage was to instil a practice of regular self-audit, in addition to generating new insights towards improving one’s practice, informing other stakeholders and sharing practice with others by way of trans-professional and transformational practice and education, inspiring action at individual, community and with collaboration, potentially wider/global levels. The reflective statement marked the end of the training programme. Clinicians then had to conform to their organisation’s consent and legal policies to practice independently and receive the final sign-off.

## Methods

### Glaucoma service clinical audit

A clinical audit was performed for the first 6 optometrists undergoing the training course, who worked at included 6 glaucoma-specialist optometrists and 240 laser procedures. All Optometrists included in the audit had already been awarded the College of Optometrists Higher Certificate in Glaucoma and were Independent Prescribers; the audit included the first ten [10] observed and ten [10] supervised procedures for each optometrist, as well as subsequent procedures performed. The audit aimed to address performance, safety and outcomes, as shown in Table 1.

**Table 1:** Clinical audit outcomes of the first 6 optometrists undergoing training.

Performance	Protocol adherence	Risks and benefit discussion Consent procedure Gonioscopy Pre- and post-laser medication, Post-laser IOP check
Safety	Intra- and post-treatment complications	
Outcomes	Clinical outcomes	Baseline IOP Post laser review IOP % IOP drop Drop reduction achieved

*IOP* intraocular pressure.

### Service evaluation

As part of an ongoing service evaluation, the views of the Glaucoma Service Director and the Lead Optometrist for Glaucoma were collected. A formal discussion with optometrists delivering SLT in the Trust also took place, mainly comparing prior experiences of training (non-structured, apprenticeship based) to the structured training programme now offered.

## Results

A total of 240 laser procedures were included in the audit; these included the in vivo training, as described above. Here we report selected results of the clinical audit and the service evaluation; detailed clinical results will be published in the future.

### Performance

A total of 100% compliance with procedures was observed, which included a risks and benefits discussion, the consent procedure and the correct prescribed post-laser medication; IOP was checked 1 h post-treatment in 99.6% of cases; 96.7% of eyes were given *g*. Apraclonidine 1% pre-treatment (subject to contra-indications) and 69.6% of the eyes had a gonioscopy performed before SLT delivery.

### Safety

Out of 240 procedures, 1 eye (0.4%) developed post-laser corneal haze and 1 eye (0.4%) developed cystoid macular oedema; both resolved with the appropriate treatment without a permanent effect on vision. A total of 9 eyes (3.7%) had an IOP spike, defined as IOP > 30 mmHg. There were no sight-threatening adverse events.

### Work-based learning resource requirements

The cost of facilitating Stages 2 and 3 (work-based learning and assessment and reflective practice) was evident for the Glaucoma Service. A total of 8 sessions per Optometrist were needed to facilitate the work-based learning and assessment (Stage 2) when Optometrists were scheduled for 1 session per week; this was the maximum that could be offered to ensure other services’ continuity. Additional logistical work included finding appropriately trained clinical supervisors for these sessions and the sign-offs. However, all optometrists who participated in the training programme were competent to be signed off following the prescribed work-based assessment schedule, with no additional training needs.

### Clinicians’ confidence

A total of 8 Optometrists employed at Moorfields Eye Hospital participated in the first round of this training programme and 7 of them are currently signed off to perform independent SLT lists in the Trust. Following the training no additional time and/or training was requested by the Optometrists, indicating self-confidence, which has been an issue of uncertainty in apprenticeship format SLT training [33]. According to the Lead Optometrist for Glaucoma, who also undertook the course, *“the course meant that the observation sessions required allowed Optometrists to focus on patient and clinic management; the opportunity to first deliver the procedure using a model eye is invaluable and takes away the fear of applying the laser, while also putting the patients at ease”*.

### Impact on ophthalmology training

Previous work has indicated that non-medical delivery of SLT needs to be approached with caution, to avoid an effect on ophthalmology training [27]. The Glaucoma Service Director at Moorfields felt confident that “*as a teaching hospital we have an obligation to train registrars and fellows, so there will always be available training sessions for those staff groups*”. Both the Glaucoma Service Director and the Lead Optometrist for Glaucoma confirmed that communication between services is key in maintaining training standards across all involved professionals and this might inevitably add some administrative workload.

## Discussion

The Moorfields/UCL laser course for ophthalmic healthcare professionals was designed following a continuously evolving field in the management of glaucoma, with calls for expansion of the professional categories treating and managing glaucoma [16–19].

Primary SLT for OAG and OHT is beneficial to patients, who can remain largely drop-free for up to 6 years after treatment [8]. Patients initially treated with SLT demonstrate a reduced need for cataract and glaucoma surgery which is a cost-effective secondary outcome for the NHS. The introduction of new SLT lists in glaucoma units in the UK is, therefore, imperative for delivering effective treatments and efficient services, but this could be at the detriment of delivering other glaucoma clinical services die to the limited supply of trained clinicians in the face of increasing demand. Reports on the glaucoma service nationally have revealed a shortage of senior ophthalmologists [17, 35], but the number of glaucoma patients is expected to grow by a fifth over the next decade and backlogs are expected to increase [17, 36]. With complex cases requiring higher level decision-making and surgical input forming approximately 20% of senior ophthalmologists’ time in the NHS [17], the need to allocate non-complex cases and procedures to other members of staff is imperative.

### Educational theory underpinning the training course

This training programme described was developed using educational principles with the aim of providing structured training to non-medical ophthalmic practitioners.

**Fig. 3:**
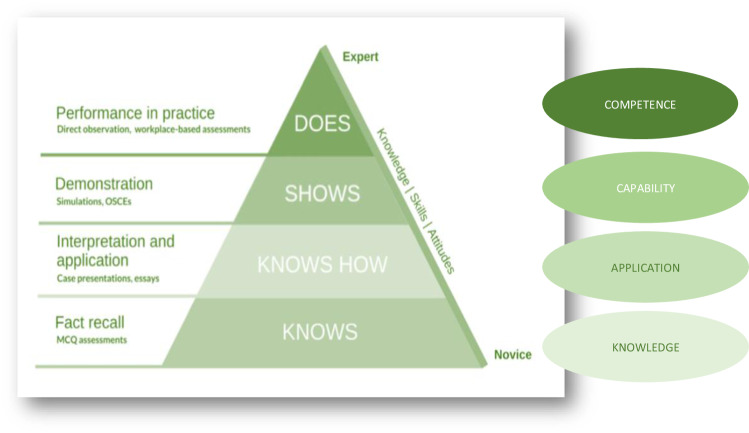
Miller’s pyramid, adapted from [44]. The levels of learning are demonstrated on the Moorfields Laser Course, depicting the development of clinical expertise through each stage of the course.

Clinical competence was ranked using Miller’s pyramid [37] (Fig. 3), which distinguishes between acquisition of knowledge and action in the workplace. In this training programme clinicians were assessed at different stages of learning leading up to the setting in which the procedure needed to be delivered. As the understanding of the clinician learners increased, they interpreted and applied knowledge taught subsequently demonstrating and performing the procedure in the workplace, ultimately reflecting the knowledge, skills and behaviours acquired. The programme was designed in three stages, to reflect a gradual build-up of knowledge, skills and confidence: Stage 1 - Knowledge acquisition and demonstration of procedure; Stage 2 - Work-based learning and assessment; Stage 3 - Reflective practice.

Clinicians were also trained on and encouraged to conduct self-audit, an important aspect of professional practice, self-evaluation and improvement, linked to self-regulated learning and self-awareness [38]. Audit improves care, safety, quality, and outcomes and can be used as an educational tool, helping to identify learning and training needs; it is recognised by the Care Quality Commission (CQC) as a professionally led exercise, which is an essential component in clinical governance and the delivery of high-quality care. The course concluded with reflective practice, described in the literature as “*a generic term for intellectual and affective activities which individuals engage to explore their experiences in order to lead to new understanding and appreciation*” [39]. In contrast to audit, reflection consolidates deep learning by reflecting on the experience, re-evaluating both negative and positive feelings of an entire learning journey and contributes to the holistic practice within a healthcare setting. At the end of this stage, learners had formed a community of practice where these experiences could be shared.

#### Structured training

A principal glaucoma-specialist optometrist regularly performing SLT, after being trained in an apprenticeship format commented on the benefits of the structured training and the exposure to model eyes commented: *“It can be quite daunting for clinicians in the early stages of training to perform a laser procedure, despite being highly trained to perform tests and make complex clinical decisions. Having a structured training process enables the clinician to grasp and embrace this new skill safely and effectively; practising on model eyes gives a feel for what happens during the procedure and what to look out for”*.

Previous qualitative data indicated that medical training could also utilise a formal, structured training framework, possibly the same as the one developed for optometrists [27]. Current ophthalmology training encompasses a laser induction course, but this is not followed by structured in vivo training. The apparent differences in the laser-training requirements between optometrists and trainee ophthalmologists may warrant further investigation and careful consideration by professional bodies. Adoption of a structured programme by ophthalmology speciality trainees and/or medical professionals trained outside the UK could be of benefit to clinicians, patients and Trusts.

#### Performance and safety of trained Optometrists

The audit performed at Moorfields Eye Hospital for 6 glaucoma-specialist optometrists having undertaken the structured course described here indicates that optometrists deliver safe treatments, whilst adhering to local protocols. IOP spikes, defined as an IOP > 30 mmHg, occurred in 3.7% of performed procedures. This is higher than 1% reported for the LiGHT trial, where an IOP spike was defined as a post-laser increase in IOP larger than 5 mmHg [8]. Differences in definitions and pre-treatment IOP are likely related to the IOP spike incidence differences between the two populations. The safety of non-medical delivery of SLT has been previously described in the UK [33] and is comparable to that reported for ophthalmologists [8, 40, 41]. However, it is worth noting that the procedure has been delivered by optometrists in the US for some years [25, 42].

#### Standardised training and governance

The lack of a formal training scheme has caused concerns around the governance of non-medically delivered ophthalmic laser procedures [27]. The main indemnity insurance provider for optometrists currently covers delivery of such procedures in the HES, assuming adequate training, accreditation and adherence to local protocols. Optometrists wishing to offer these services outside the HES have other indemnity insurance options.

Our previous work identified the lack of standardised training as a challenge in developing a healthcare system supportive of ophthalmic practitioners competent in delivering ophthalmic laser treatments. The training programme described here was developed to address this concern and was the first UK hospital/university based structured laser training available to ophthalmic practitioners. Recently one more UK university has developed a similar programme for eye-care practitioners [43], indicating a need for a national approach to the training of non-medical ophthalmic practitioners in laser procedures. The current availability of two UK ophthalmic laser-training courses and the significant increase in the number of optometrists delivering SLT in the HES [20] calls for the need to develop national standardised training and treatment protocols.

## Conclusions

The delivery of glaucoma care is changing fast, with more non-medical ophthalmic practitioners successfully and efficiently involved in the care of patients diagnosed with glaucoma. In the UK, as most glaucoma units actively involve non-medical ophthalmic practitioners in enhanced and advanced roles, formal and structured training is needed to standardise the quality of ophthalmic laser treatments delivered; such a training programme can potentially be applicable to all professionals delivering SLT Here we propose an education and training programme for the delivery of SLT by non-medical ophthalmic practitioners. Early results indicate that trained optometrists follow the relevant clinical protocols and deliver safe laser treatments. Despite an initial investment of time, trained optometrists can take on a significant clinical load, working autonomously. Further publications on the efficacy of optometrist-delivered SLT will follow, aiming to enrich the evidence base of non-medical ophthalmic laser delivery.

## Summary

### What was known before

- SLT is as effective as eyedrops in controlling IOP in eyes with open-angle glaucoma or ocular hypertension. Almost 70% of eyes remain drop-free for at least 6 years after primary treatment with SLT. SLT has been shown to delay the need for surgery to lower IOP. SLT is also cost-effective for the NHS and is now the 1st line treatment for glaucoma and ocular hypertension.

### What this study adds

- Here we present the design and delivery of a laser-training programme for ophthalmic practitioners The programme is based on medical education principles This work addresses the current lack of a standardised laser-training course for non-medical professionals and the sharp increase in professionals needed to deliver this treatment in the HES.

## Data Availability

The data that support the findings of this study are located in Moorfields Eye Hospital and the Institute of Ophthalmology, UCL and are available from the corresponding author upon reasonable request.
